# Comparative Study of Donepezil-Loaded Formulations for the Treatment of Alzheimer’s Disease by Nasal Administration

**DOI:** 10.3390/gels8110715

**Published:** 2022-11-05

**Authors:** Lupe Carolina Espinoza, Diana Guaya, Ana Cristina Calpena, Rodolfo Miguel Perotti, Lyda Halbaut, Lilian Sosa, Adriel Brito-Llera, Mireia Mallandrich

**Affiliations:** 1 Departamento de Química, Universidad Técnica Particular de Loja, Loja 1101608, Ecuador; 2 Institute of Nanoscience and Nanotechnology (IN2UB), University of Barcelona, 08028 Barcelona, Spain; 3 Department of Pharmacy, Pharmaceutical Technology and Physical Chemistry, Faculty of Pharmacy and Food Sciences, University of Barcelona, 08028 Barcelona, Spain; 4 Pharmaceutical Technology Research Group, Faculty of Chemical Sciences and Pharmacy, National Autonomous University of Honduras (UNAH), Tegucigalpa 11101, Honduras; 5 Dirección de Ciencia, Tecnología e Innovación, Universidad de la Habana, Calle M entre 19 y 21 #255, Vedado, La Habana 10400, Cuba

**Keywords:** Alzheimer’s disease, gel, intranasal administration, donepezil

## Abstract

Alzheimer’s disease is characterized by a progressive deterioration of neurons resulting in a steady loss of cognitive functions and memory. Many treatments encounter the challenge of overcoming the blood–brain barrier, thus the intranasal route is a non-invasive effective alternative that enhances the drug delivery in the target organ–the brain–and reduces the side effects associated with systemic administration. This study aimed at developing intranasal gels of donepezil as an approach to Alzheimer’s disease. Three different gels were elaborated and characterized in terms of pH, morphology, gelation temperature, rheology, and swelling. An in vitro release study and an ex vivo permeation in porcine nasal mucosa were conducted on Franz diffusion cells. The tolerability of the formulations was determined by the cytotoxicity in human nasal cells RPMI 2650. Results showed that pluronic gels exhibit the higher release rate and enhanced permeation compared to chitosan gel. Moreover, the combination of Pluronic F-127 and Transcutol^®^ P exerted a synergic effect on the permeation of donepezil through the nasal mucosa. The resulting gels showed suitable tolerance in the RPMI 2650 cell line and physicochemical characteristics for intranasal delivery, and thus gel formulations administered by nasal mucosa could be an alternative strategy to improve the bioavailability of donepezil.

## 1. Introduction

Neurological diseases have become a common health problem worldwide. Some neurological health problems are promoted by genetics, nutrition, and lifestyle aspects [1]. Alzheimer’s disease (AD) is a chronic affection of neurons of the brain and nervous system that cause dementia. The etiology of AD is not fully known; however, the neurons’ function is progressively deteriorated which promotes a deficit in the memory, abrupt changes in behavior, and cognitive impairment. Every day–worldwide–there are a greater number of cases associated with AD; thus, it is a public health problem that deserves attention since it is greatly impacting society [2].

**Figure 1:**
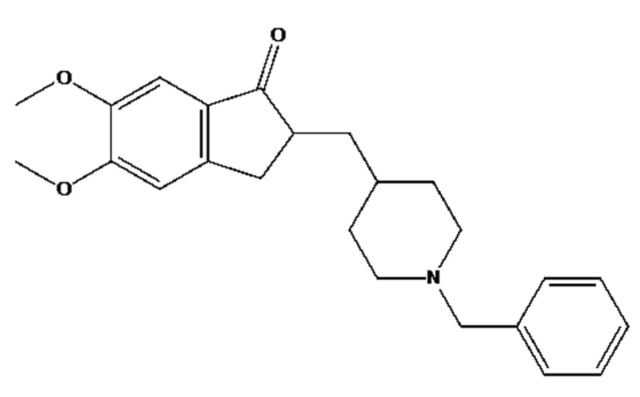
Chemical structure of Donepezil.

Many studies have been performed to find effective treatments for this disease, however there are not curative treatments for AD since only symptomatic treatments have been available up till now [3]. Conventionally, the existing treatments take into account: (i) to re-establish the cholinergic function inhibiting the acetylcholinesterase enzyme activity, and (ii) to reduce the glutamate excitotoxicity through the antagonism action of N-methyl-D-aspartate receptors (NMDAr) [4,5]. Cholinergic deficit has been reported to be the major pathological cause for AD. In this case, the use of some acetylcholinesterase (AChE) inhibitors including galantamine, risvastigmine, and donepezil hydrochloride (DPZ) have been used to improve cholinergic function. DPZ is a second-generation drug used for the treatment of neurodegenerative disease that works via the impairment of cholinergic neurotransmission [6,7]. Figure 1 shows the chemical structure of DPZ.

The effectiveness of the treatment of brain pathologies is complex, and it becomes a challenge that is conditioned by drug administration. The role of the blood–brain barrier (BBB) limits the entry and distribution of drugs to the brain [1]. However, the nasal pathway allows the delivery of drugs directly to the brain, bypassing the BBB. Nasal administration avoids first-pass liver metabolism and minimize adverse reactions since the drug distribution can reach the targeted sites [8]. However, the intranasal drug administration technique has some limitations such as the enzymatic degradation and low drug residence time by effect of the nasal mucociliary clearance system. Thus, the use of mucoadhesive polymers have been used to enhance the drug retention times and mucosal permeation, improving their bioavailability [9].

Synthetic and natural polymers including chitosan and poloxamers are used as excipients of nasal drug administration systems [10]. Chitosan is a biodegradable polysaccharide obtained from chitin, which mainly originates from crustacean shells. Chitosan is a biocompatible and non-harmful material, and it has been widely used in drug formulation for nose-to-brain (N2B) delivery routes. Chitosan has demonstrated to provide longer retention times of drug formulations in the nasal cavity, improving the hydrophilic molecules absorption and permeation through the nasal epithelial cell membrane [11]. On the other hand, the most used temperature responsive polymers are poloxamers such as Pluronic block copolymers, which have been used for brain delivery drugs. Pluronic F-127 is a commercial polymer that forms gel in situ due to the increase in temperature; it is suitable for the development of formulations at ambient nasal temperatures. The gels prepared from Pluronic F-127 demonstrated being an effective mucoadhesive agent that prolongs the drug retention time at body temperatures with a delayed nasal mucociliary clearance and increase in brain uptake of the drugs [1].

In our previous work, we reported the development of nanoemulsions (NE) containing DPZ as a therapeutic agent for symptoms of Alzheimer’s [12]. Non-invasive techniques are desirable for enhancing the nose-to-brain drug release and absorption that is suitable for geriatric patients and in advanced stages of the disease. However, to achieve treatment success using the nasal route, it is necessary to incorporate formulation strategies to improve the drug’s solubility and permeability, as well as to extend the residence time of the formulation at the site of application. To this end, gel formulations using different mucoadhesive polymers such as chitosan and Pluronic F-127 could increase the viscosity and, in turn, the residence time at the absorption site, potentially resulting in increased drug bioavailability. Thus, the aim of this work was to develop gel formulations with chitosan and Pluronic F-127 loaded with DPZ as an approach in the development of Alzheimer’s disease treatment by nasal administration. The addition of a safe and biocompatible permeation enhancer was also considered.

## 2. Results and Discussion

### 2.1. Solubility and Preparation of DPZ Gels

**Figure 2:**
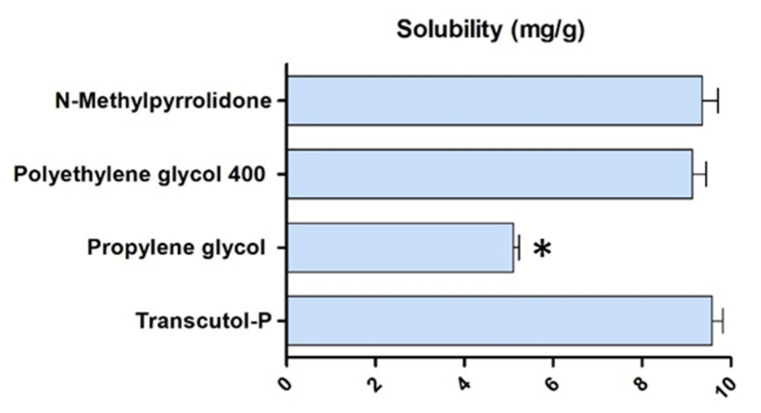
Solubility of DPZ in different cosolvents. * Statistically significant differences with N-methylpyrroline, polyethylene glycol 400, and Transcutol-P; statistical significancy set at *p* < 0.05.

Figure 2 shows the solubility results of the cosolvents tested for DPZ. The water-soluble cosolvents with the highest solubilizing potential for DPZ were Transcutol^®^ P and N-Methylpyrrolidone (NMP), and were consequently used for gel formulations. Transcutol^®^ P is a widely used solvent in the development of pharmaceutical and cosmetic products due to its high biocompatibility, non-toxic properties, and solubilizing potential of both lipophilic and hydrophilic drugs [13]. N-Methylpyrrolidone (NMP) is a polar solvent frequently used in the development of pharmaceutical products. NMP has a safety profile at established doses and increases the solubility and permeability of multiple drugs [14].

The composition of the three DPZ-loaded gels obtained in this study are detailed in Table 1. For DPZ-CGel preparation, chitosan (1%) was dissolved in 0.1 M aqueous acetic acid solution (20%) and then water (59%) was added to this mix. DPZ (6.25 mg/mL) was solubilized in Transcutol^®^ P (20%) and then incorporated to the chitosan solution. For thermoreversible formulations DPZ-PGel_1_ and DPZ-PGel_2_, Pluronic F-127 (18%) was dissolved in cold purified water and then DPZ (6.25 mg/mL) previously solubilized in Transcutol^®^ P (20%) or N-Methylpyrrolidone (5%) was added to the polymer solution, respectively (Table 1).

### 2.2. Physicochemical Characterization of DPZ-CGel, DPZ-PGel_1_ and DPZ-PGel_2_

The three obtained gels showed a transparent and homogeneous appearance. They registered a pH value of 5.9, 6.2, and 6.3 for DPZ-CGEL, DPZ-PGel_1,_ and DPZ-PGel_2_, respectively. The pH value determined is optimal for intranasal drug administration since it is compatible with the epithelium and ciliary system that requires a pH in the range of 5.0 to 6.5, suggesting that the gels obtained would not cause irritation to the nasal cavity [2,8].

**Figure 3:**
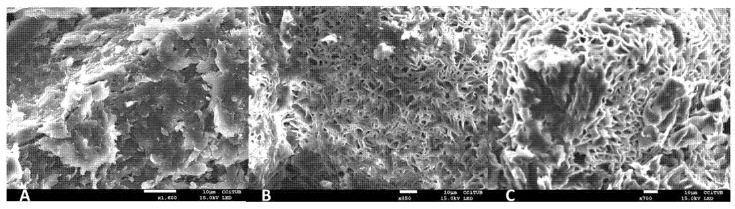
Scanning electron microscopy (SEM) images of (**A**) DPZ-CGel magnification 850×, (**B**) DPZ-PGel_1_ and (**C**) DPZ-PGel_2_ magnification 450×. Scale bar equals 10 µm.

The scanning electron microscopy (SEM) images revealed the morphology of the three DPZ-loaded gels (Figure 3). DPZ-CGel exhibited a structure more compact in comparison to the pluronic formulations (DPZ-PGel_1_ and DPZ-PGel_2_), which showed a porous structure with interconnected capillary channels. Although the pluronic formulations showed a very similar gel network structure, it can be observed that DPZ-PGel_1_ seems to have a more uniform network of fibers than the DPZ-PGel_2_, which is attributed to the solvents used Transcutol^®^ P and N-Methylpyrrolidone for DPZ-PGel_1_ and DPZ-PGel_2_, respectively.

The thermoreversible properties of DPZ-PGel_1_ and DPZ-PGel_2_ were confirmed, showing a gelation temperature of 32 and 33 °C for DPZ-PGel_1_ and DPZ-PGel_2_, respectively. These characteristics are ideal for nasal administration since they show a liquid form for easy application that turns into a gel when in contact with the nasal cavity.

**Figure 4:**
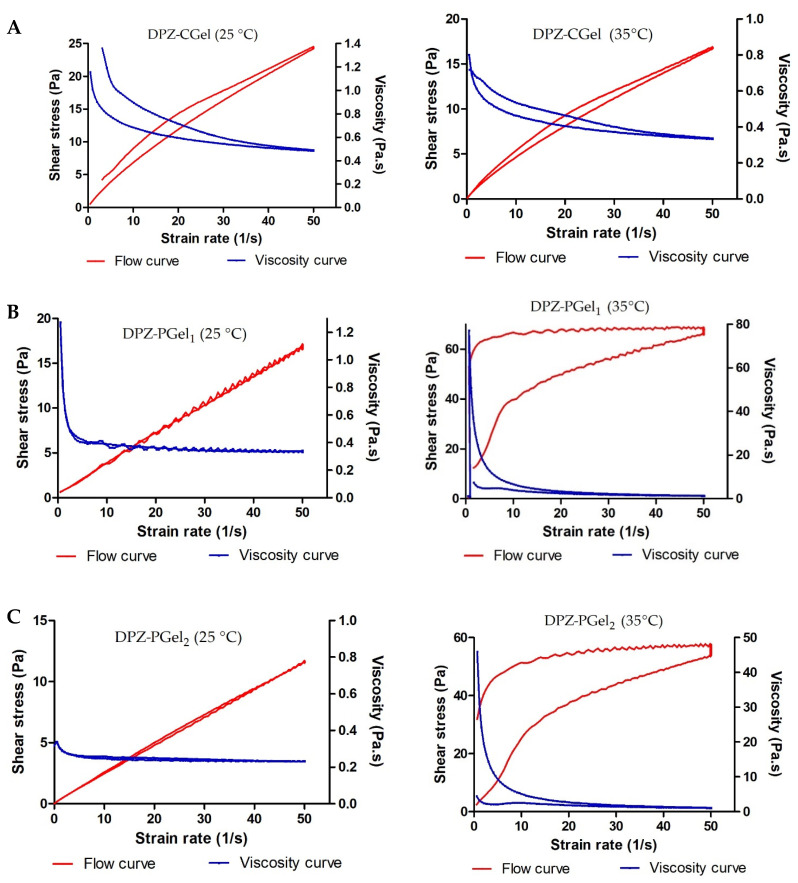
Flow and viscosity curves at 25 °C and 35 °C for (**A**) DPZ-CGEL, (**B**) DPZ-PGel_1_ and (**C**) DPZ-PGel_2_.

The rheological behavior determines sensory properties, filling/dosing characteristics, diffusibility, and modulates biopharmaceutical parameters including the release rates of the drug from its carrier [15,16]. The rheological behavior of the three DPZ formulations developed in this study is shown in Figure 4. DPZ-CGel revealed the highest viscosity compared to DPZ-PGel_1_ and DPZ-PGel_2_ at 25 °C. However, when the temperature increased to 35 °C, the pluronic formulations (DPZ-PGel_1_ and DPZ-PGel_2_) demonstrated a higher viscosity than the chitosan formulation (DPZ-CGel). DPZ-CGel showed at 50 s^−1^ an average viscosity of 487 ± 1.5 mPa·s at 25 °C, whereas it became more fluid with an increase in temperature, showing a value of 335 ± 0.9 mPa·S at 35 °C. As expected, the rheological behavior of DPZ-PGel_1_ and DPZ-PGel_2_ depended on the temperature due to the thermoreversible properties of these poloxamer-based gels (Figure 4B,C): at lower temperatures, they are more fluid, thus facilitating their administration by a spray; once they come into contact with the organism at corporal temperature, the solubility of the PPO (polypropylene oxide) chains decreases, forming micelles and cubic and hexagonal structures and thus increasing the viscosity. This thermoreversibility could help reduce dripping delaying mucociliary clearance and improving drug retention in the application site [16,17,18].

The experimental data were fitted to mathematical models and the rotational testing results are summarized in Table 2. Rheological analysis and mathematical modeling confirmed the shear-thinning (pseudoplastic) behavior of all formulations at both temperatures, which was expected in this type of formulation. Likewise, the presence of a hysteresis loop in the rheograms was demonstrated, suggesting an apparent thixotropic behavior in the three DPZ formulations. Thixotropy has advantages above all because it facilitates the application of the formulations–since their viscosity varies with friction [19].

**Figure 5:**
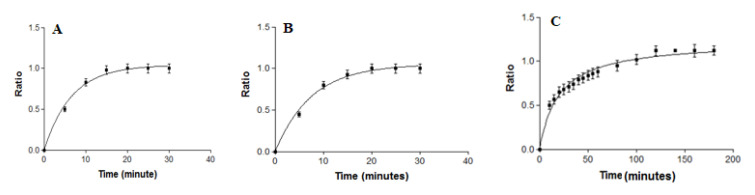
Swelling ratio versus time of Donepezil formulations (**A**) DPZ-PGel_1_, (**B**) DPZ-PGel_2_, (**C**) DPZ-CGel.

The swelling process of DPZ-PGel_1_ and DPZ-PGel_2_ followed a first-order kinetic model, represented by the kinetic constant (k) of 0.15 min^−1^ and 0.12 min^−1^, respectively (Figure 5A,B). On the other hand, the percentage of porosity for DPZ-PGel_1_ and DPZ-PGel_2_ was 80.90 ± 2.19% and 81.34 ± 1.15%, respectively. In addition, in both cases the equilibrium process was completed at 20 min, which is considered a normal behavior for this type of system; likewise, the percentages of porosity ranged between 79 and 82% [20,21]. The porosity of DPZ-PGel_1_ and DPZ-PGel_2_ can also be seen through the images obtained by SEM (Figure 3).

The swelling of DPZ-CGel increased rapidly in the first hours, which could be explained by the large number of free adsorption sites on the surface of the pores of the gel. The swelling then decreased until equilibrium was reached. The mathematical behavior shown was through the hyperbola model with a constant kinetics (k) of 20.30 min^−1^ (Figure 5C). This behavior of chitosan gels, in terms of swelling, has been previously reported by other authors [22,23]. Regarding the percentage of porosity, this gel presented 82.05 ± 2.97%; this porosity percentage is similar to that found by Ikeda and coworkers [24].

### 2.3. Stability Studies

The stability studies of DPZ formulations showed no significant changes in its appearance after 30 days of storage at 25 and 40 °C (Table 3 and Table 4). In the same way, pH values remained within the range suitable for nasal administration. Finally, the content of drug remained without significant changes at 25 and 40 °C over a span of 30 days of study; DPZ-CGel showed further degradation at 40 °C with a decrease of 1.03% compared with the pluronic formulations. Therefore, an acceptable physical and chemical stability with slight yet insignificant changes is suggested, demonstrating compatibility between the drug and the excipients.

### 2.4. Cytotoxicity Results

**Figure 6:**
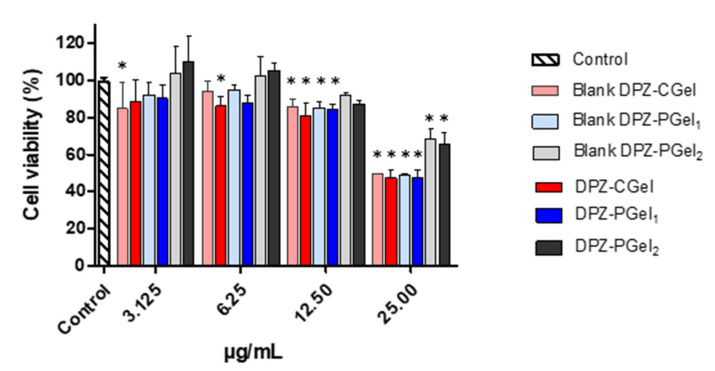
In vitro cytotoxicity of donepezil (DPZ) formulations. Although statistical differences to the control were observed at different concentrations, cells were viable up to 12.5 µg/mL. * Statistical significance set at *p* < 0.05.

The tolerability of the formulations was analyzed by cytotoxicity studies using human nasal cell line RPMI 2650 in order to simulate real nasal mucosa [25,26]. No apparent cytotoxicity was observed in the three formulations since the cell viability was above 80% within the dilutions ranging from 3.125 to 12.5 µg/mL for chitosan and Pluronic F-127 formulations (Figure 6).

### 2.5. In Vitro Release Studies

**Figure 7:**
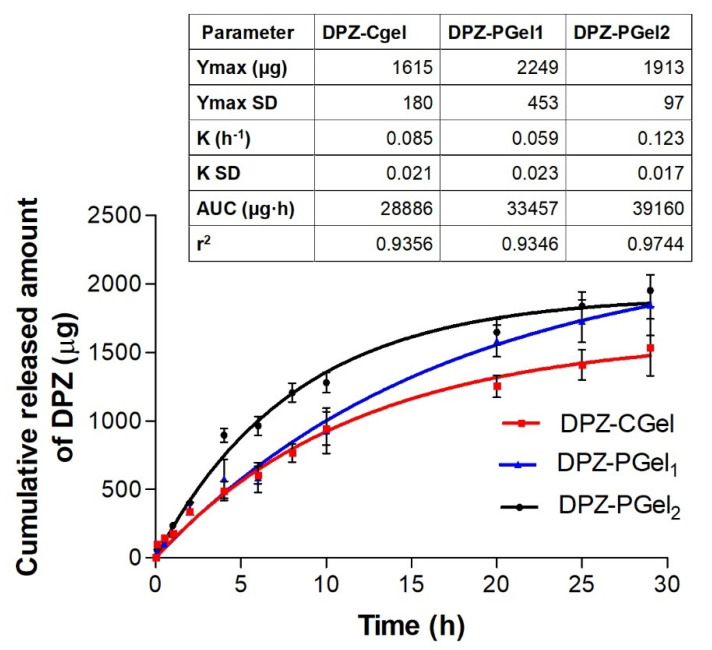
Profiles of donepezil released from the formulations, assessed by in vitro drug release tests.

The in vitro drug release of the formulations with chitosan and Pluronic F-127 was investigated by Franz cells using a dialysis membrane. The release profile of the DPZ formulations are depicted in Figure 7. At the end of the experiment, DPZ-PGel_1_ and DPZ-PGel_2_ released amounts greater than 98% of the drug, whereas DPZ-CGel released 81.8% of the drug incorporated into the formulation, confirming that all formulations released the drug they contained. First-order kinetic was the model that best fit the data, which was selected according to the coefficient of determination (r^2^) for the three formulations. First-order kinetics describes the release process as a Fickian diffusion in which the release rate of DPZ is in proportional relation to the amounts of drug remaining in the vehicle [20]. The maximum release amount (Ymax) of 1615, 2249, and 1913 µg for DPZ-Cgel, DPZ-PGel_1,_ and DPZ-PGel_2_, respectively.

### 2.6. Ex vivo Permeation Studies

**Figure 8:**
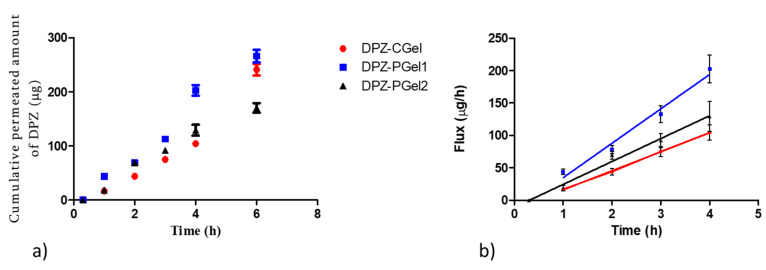
(**a**) Permeation profile of donepezil (DPZ) from formulations through ex vivo nasal mucosa; (**b**) Estimated flux or permeation rate of DPZ, calculated as the slope of the linear part of the permeation profile by linear regression analysis.

The permeation profile of DPZ through nasal mucosa is depicted in Figure 8a. Chitosan and pluronic formulations were tested, DPZ-PGel_1_ showed a higher slope, which means that it has a better flux or permeation rate compared with DPZ-CGel and DPZ-PGel_2_ (Figure 8b). This finding was confirmed with biopharmaceutical analysis, shown in Table 5.

Table 5 shows the biopharmaceutical parameters calculated in the permeation study of chitosan and Pluronic F-127 formulations. DPZ-Pgel_1_ showed higher values of flux (J_ss_), permeability coefficient (K_p_), partition coefficient vehicle/tissue (P_1_), diffusion coefficient (P_2_), and theoretical plasma concentration at the steady-state in humans (C_ss_). The lag time (Tl) was similar around 20 min for the three formulations. Additionally, DPZ-PGel_1_ exhibited a higher retention of DPZ into the nasal mucosa (Qret) than DPZ-CGel and DPZ-PGel_2_.

Although DPZ-PGel_2_ showed the highest release rate (Figure 7), the ex vivo permeation studies using porcine nasal mucosa indicated better results for DPZ-PGel_1_, which is attributed to Transcutol^®^ P content possibly due to its permeation enhancing properties. The permeability of a given drug depends on both the physicochemical properties of the drug and the affinity of the drug to the tissue. The parameters P_1_ and P_2_ provide information about what mechanism is the driven force of the permeation. In both gels, P_1_ is higher than P_2_, meaning that the partition between the vehicle and the nasal mucosa has a greater impact on the permeation of DPZ through the nasal mucosa. When comparing P_2_ (diffusion coefficient) between DPZ-PGel_1_ and DPZ-PGel_2_, both gels have similar values; however, P1 for DPZ-PGel_1_ is nearly 3-fold DPZ-PGel_1_‘s value, suggesting that Transcutol^®^ P increases the affinity of the drug to the nasal mucosa, resulting in an enhanced permeation of DPZ. Apparently, Pluronic F-127 exerted a synergic effect on the permeation of DPZ, since both gels DPZ-CGel and DPZ-PGel_1_ contained Transcutol^®^ P in their composition, a similar pH, and differed in the gelator used–either chitosan or pluronic.

According to the lag time (Tl), the drug would reach the steady-state in about 20–30 min with no significant statistical differences between DPZ-PGel_1_ and DPZ-PGel_2_, whereas DPZ-CGel takes longer to reach the steady-state. DPZ-PGel_1_ showed the greatest partition coefficient (P_1_), indicating that the driving force of DPZ permeation through the nasal mucosa is due to the partition formulation-mucosa. The chitosan-based gel (DPZ_CGel) showed significant statistical differences with pluronic-based gels (DPZ-PGel_1_ and DPZ-PGel_2_) for all the permeation parameters. DPZ-PGel_1_ and DPZ-PGel_2_ only showed statistical differences between them for flux, P_1_, Kp, and C_ss_. In other words, J_ss_, K_p_, P_1_, and C_ss_ present statistical differences between all the formulations–with DPZ-PGel_1_ having the greatest values and DPZ-CGel the lowest values. Concerning the retained drug amount in the nasal mucosa, no significant statistical differences were found between pluronic-based gels; hence, they would have similar depot effects. The plasma concentration at the steady-state (C_ss_) predicted that pluronic-based gels would have systemic effect after intranasal administration. Therefore, intranasal delivery could be an alternative route to oral administration, using less than half of the oral dose in a single dose versus oral repeated doses, and consequently reducing the side effects. These results are in line with those obtained in a previous work in which we formulated DPZ in nanoemulsions for intranasal delivery [27]. Other researchers have also investigated the advantages of the intranasal route by the nose-to brain delivery of DPZ–for instance, Rajput et al. prepared a gel loading DPZ liposomes for the intranasal administration and compared it to DPZ orally administered; they observed that DPZ was mainly distributed in the brain from the intranasal administration and the opposite for the oral administration, in which DPZ was mainly located in the plasma [28]. Gangopathyay and co-workers evaluated the permeation of DPZ gel in ex vivo nasal mucosa from sheep, resulting in an increased permeation of DPZ [29]. High permeation was also observed by Gangane et al. of DPZ microspheres gel in ex vivo goat and sheep nasal mucosa [30,31]. Patil R.P tested a gel of cubosomes containing DPZ administered intranasally to rats, and they studied the biodistribution of DPZ in brain tissue and plasma. They observed a higher permeation through the nasal mucosa from the cubosomes formulations as well as a higher distribution of DPZ in the brain than the plain solution [28,32]. Based on these promising results, further pre-clinical studies are considered. On one hand, a deeper characterization of the formulations would be worthwhile in order to study the degradation kinetics of the gels as well as evaluating the mucoadhesivity of the gels on ex vivo porcine nasal mucosa. On the other hand, determining the amount of DPZ in the brain tissue using in vivo models is being also considered in order to explore, in future works, the use of these gel formulations as alternatives to improve the efficacy of the drug.

## 3. Conclusions

DPZ- loaded gels with two different polymers (chitosan and Pluronic F127) have been developed. The gelation temperature of pluronic-based gels was stablished at about 32.5 °C, which is ideal for nasal administration since it can be administered in liquid form and turns into a gel when in contact with the nasal cavity. These thermoreversible gels exhibited the higher release rate and enhanced permeation compared to the chitosan formulation. Additionally, the use of Transcutol^®^ P as a solvent maximized the drug permeation through the porcine nasal mucosa. The three formulations showed to be stable at 30 and 40 °C. The gels were well tolerated since the cell viability in human nasal RPMI 2650 cells was satisfactory up to 12.5 µg/mL.

## 4. Materials and Methods

### 4.1. Materials

The drug (DPZ) was acquired from Capot Chemical (Hangzhou, China). Diethylene glycol monoethyl ether (Transcutol^®^ P) was supplied by Gattefossé (Saint-Priest, France), Pluronic F-127 (Poloxamer 407) was purchased from Fagron (Barcelona, Spain). Deacetylated chitin (Chitosan) and the RPMI 2650 cell lines were obtained from Sigma-Aldrich (Darmstadt, Germany). Reagents related to MTT assay were acquired from Gibco (Carcavelos, Portugal) and Invitrogen Alfagene (Carcavelos, Portugal). Reagents used in the analytical method and sample analysis were acquired from Panreac (Barcelona, Spain); a Millipore Milli-Q purification system (Millipore Corporation, Burlington, MA, USA) provided the purified water used in this work.

### 4.2. Solubility Studies

In order to select the cosolvents of the formulations, the solubility of DPZ was evaluated in different excipients including Transcutol^®^ P, propylene glycol, polyethylene glycol 400, and N-Methylpyrrolidone. An amount of 15 mg of drug was mixed with 2 g of each of these cosolvents under magnetic stirring during 120 min at 25 °C. These samples were equilibrated for 12 h at room temperature. Then, they were centrifuged at 9000 rpm for 15 min, and the supernatant was isolated and diluted using a mixture of methanol:water (50:50, *v*/*v*). Finally, the drug was quantified by DR 6000 UV-Visible Spectrophotometer (Hach, Düsseldorf, Germany) at 312 nm.

### 4.3. Preparation of Formulations

For polymer solutions, chitosan was dissolved in 0.1 M of acetic acid solution (DPZ-CGEL) and Pluronic F-127 was dissolved in cold purified water (DPZ-PGel_1_ and DPZ-PGel_2_) under magnetic stirring for 30 min. DPZ was solubilized in the cosolvents that exhibited greater capacity to solubilize the drug and then was immediately incorporated in the corresponding polymer solution under the same stirring conditions for 10 min.

### 4.4. Physicochemical Characterization of DPZ-CGel, DPZ-PGel_1_ and DPZ-PGel_2_

#### 4.4.1. pH Determination

A digital pHmeter (Crison, pH & ION- Meter GLP 22, Spain) at 25 °C was used to determine the pH of DPZ-CGEL, DPZ-PGel_1_, and DPZ-PGel_2_.

#### 4.4.2. Morphological Analysis

To analyze the morphology, samples (5 g) of each formulation (DPZ-CGEL, DPZ-PGel_1_, and DPZ-PGel_2_) were dried until constant weight using a vacuum desiccator. A sample of dry gel was examined by Scanning Electron Microscopy (SEM) by carbon coating layer using a JEOL J-7100F (JEOL Inc., MA, USA).

#### 4.4.3. Gelation Temperature

The gelation temperature was evaluated using an aliquot (2 mL) of the pluronic formulations (DPZ-PGel_1_ and DPZ-PGel_2_) contained on a glass vial, which were immersed in a water bath at 12 °C. The temperature of water bath had increments of 1 °C and equilibration times of 3 min for each new setting. After the vial was rotated 180°, the gelation point was established at stable meniscus.

#### 4.4.4. Viscosity and Rheological Behavior

The rheological behavior was evaluated using a Thermo Scientific Haake Rheostress 1 rotational rheometer (Thermo Fisher Scientific, Karlsruhe, Germany). Curves of viscosity and flow were determined initially at 25 °C and then at 35 °C, 24 h after preparation for DPZ-CGEL, DPZ-PGel1, and DPZ-PGel2 formulations (n = 2). The test conditions included a ramp-up period from 0 to 50 s^−1^ for 3 min, a constant shear rate period of 50 s^−1^ for 1 min, and a ramp-down period from 50 to 0 s^−1^ for 3 min. The obtained data from the flow curve (shear stress (τ) versus shear rate ($\dot{\gamma }$)) were fitted to the mathematical models described in Table 6 in order to establish which best statistically represents the experimental data according to the correlation coefficient value (r). On the other hand, the determination of the disturbance of the microstructure during the test or “apparent thixotropy” (Pa/s) was evaluated by determining the area of the hysteresis loop.

#### 4.4.5. Swelling Test

For the swelling tests (ST), an amount of 0.5 g of dried gels was used. The samples were incubated in PBS buffer (pH = 5.5) at a temperature of 32 °C for 30 min for Pluronic F-127 gels (DPZ-PGel_1_ and DPZ-PGel_2_). In the case of the chitosan gel (DPZ-CGel), the study was carried out for approximately 3 days. The samples were weighed after drying excess water at predetermined time intervals. Assays were performed in triplicate (n = 3).

The swelling ratio (*SR*) value was determined with the equation 1. In addition, the results were fitted to a kinetic model:

(1) $$SR=\frac{W_{s}-W_{d}}{W_{d}}$$

where *W_s_* is the weight of the swollen gel at the intervals of 5 min (Pluronic F-127 gels) and 1 h (Chitosane gel), and *W_d_* is the weight of the dry gel.

The percentage of porosity (*P*) was calculated by the method known as solvent displacement. The previously dried gels (DPZ-CGel, DPZ-PGel_1_, and DPZ-PGel_2_) were weighed and then immersed in absolute ethanol for 2 min, then the excess ethanol on the surface was dried and weighed again. The percentage of porosity was determined using the following equation.

(2) $$P=\frac{W_{2}-W_{1}}{\rho \;\times \;V}$$

where, *W*_1_ is the initial weight of the dry gel, *W_2_* is the weight of the gel after being immersed in ethanol, ρ is the absolute ethanol density, and V is the gel volume.

### 4.5. Stability Assays

The physical and chemical stability of the three formulations DPZ-CGEL, DPZ-PGel_1,_ and DPZ-PGel_2_ was studied by the evaluation of parameters such as appearance, pH, and drug content at 25 and 40 ◦C for 30 days. A Waters High-Performance Liquid Chromatography (HPLC) with 2487 (UV/Vis) Detector & 717 Plus Autosampler (Waters, Milford, MA, USA) was used to determine the drug content. This analysis was performed using a Kromasil C_18_ column (250 × 4.6 mm × 5 μm). A solution of methanol:buffer (50:50, *v*/*v*) was used as mobile phase (MP). The buffer at pH 2.5 was prepared using a solution of potassium dihydrogen orthophosphate 0.05 M, 5 mL of trimethylamine. The pH was adjusted using orthophosphoric acid. This MP was pumped through the Kromasil column at a flow rate of 1.2 mL/min. A volume of 20 μL was injected, and the elute was analyzed at 268 nm. Data were processed using Empower 3 software—Build 3471 (Waters, Milford, MA, USA, 2010).

### 4.6. Cytotoxicity Assay

The Methylthiazolyldiphenyl-tetrazolium bromide (MTT) cytotoxicity assay was carried out using the human nasal cells RPMI 2650 (Sigma Aldrich). These cells (2 × 10^5^ cells/mL) were cultured in 96-well cell culture plates (Corning Inc., Corning, NY, USA) and maintained at 37 °C and 5% CO_2_ atmosphere in an incubator for 24 h. The Eagle’s minimum essential medium (EMEM) was used for cells growth and complemented with 2 mM glutamine, 1% non-essential amino acids (NEAA), 100 U/mL penicillin, 100 μg/mL streptomycin, and 10% heat inactivated fetal bovine serum (FBS). DPZ-CGel, DPZ-PGel_1_ and DPZ-PGel_2_ were diluted at several concentration of drug from 3.125 to 25 μg/mL and were added to the cell cultures for 24 h. Blank gels were used for comparison. Afterwards, the medium was removed, and the nasal cells were incubated with fresh medium containing 10% of MTT (5 mg/mL in phosphate buffered) at 37 °C for 2 h. Again, the medium was removed, and the nasal cells were exposed to 100 μL of dimethyl sulfoxide (DMSO, 99%) in order to lysate the cells and dissolve the MTT crystals. These cells were transferred to a new 96-well cell culture plates and the absorbance was determined at an excitation/emission of 540/630 nm using a Microplate Autoreader (Modulus Microplate Multimode Reader-Turner Biosystems, Sunnyvale, CA, USA). In addition, a negative control (cells without any treatment) was assayed for comparison using the same method. The cell viability was determined considering the direct proportions to the absorbance values.

### 4.7. In Vitro Drug Release Study

The drug released from the formulations DPZ-CGEL, DPZ-PGel_1,_ and DPZ-PGel_2_ was studied using Franz-type diffusion cells of 6 mL (Vidra Foc, Barcelona, Spain) with an effective diffusion area of 0.64 cm^2^. The receptor medium (RM) consisted of methanol:Transcutol^®^P (1:1, *v*/*v*), which provided the sink conditions during the whole study along with a stirring rate of 100 rpm. The receptor medium thermostated at 37 ± 0.5 °C and a dialysis membrane composed of cellulose of 12–14 kDa pore size (Merck, Spain) was placed between the donor and receptor compartment. Next, 0.3 mL of the formulation was applied to the dialysis membrane, and samples were collected at different time points for 29 h (0.08, 0.5, 1, 2, 4, 6, 8, 10, 20, 25, and 29 h). The DPZ released from the three formulation was determined by HPLC (see Section 4.5). The amount of DPZ released was plotted as a function of time (mean ± standard deviation of three replicates), and the kinetic profile of DPZ released was fitted to five kinetic models described in Table 7. The best-fitted model profile was selected, taking into account the r^2^ value ≈ 1.

### 4.8. Permeation Studies through Ex Vivo Nasal Mucosa

The permeability of DPZ through nasal mucosa was evaluated on ex vivo porcine nasal mucosa dermatomed at a thickness of 500 ± 100 µm. The tissue was excised from pigs from the Animal Facility of the Faculty of Medicine under the approval of the Ethical Committee of Animal Experimentation of the University of Barcelona, Spain (CEEA-UB). The experiment was performed on Franz cells (FDC 400; Crown Grass, Somerville, NJ, USA) of 6 mL of capacity and 0.64 cm^2^ of diffusion area. The RM was Transcutol^®^P:water (60:40, *v*/*v*), which was maintained at 37 ± 0.5 ◦C and 100 rpm. A sample of 0.3 mL of DPZ-gel was applied to the nasal mucosa, and aliquots of 0.3 mL of RM were collected at different time intervals and replaced with fresh RM (0.3, 1, 2, 3 and 4 h). The amount of DPZ in the RM samples was quantified according the HPLC methodology previously described (Section 4.5), corresponding to the amount of DPZ that had permeated through the nasal mucosa. Six replicates for each formulation were included in the experiment, and results are expressed as mean ± SD and plotted as the cumulative amount of drug permeated (µg) as a function of time (h) to obtain the permeation profile. The following biopharmaceutical parameters were estimated: the flux (Jss, µg/(h/cm^2^)), which corresponds to the permeation rate at the steady-state and was calculated as the slope of the linear part of the permeation profile by linear regression analysis; the permeability coefficient (K_p_, (cm/h)) was also calculated, as well as the lag time (TL, h), partition coefficient between vehicle and tissue (P_1_, cm) and the diffusion coefficient (P_2_, h^−1^) [10]. Additionally, the theoretical concentration that would be achieved in plasma after the nasal administration of DPZ-gels in human (*C*_*ss*_) was also predicted by means of Equation (3).

(3) $$C_{ss}=J_{ss}\;\frac{A}{{Cl}_{p}}$$

where *J*_*ss*_ is the flux at the steady-state, *A* is the hypothetical area of application (150 cm^2^ for nasal mucosa), and *Cl*_*p*_ is the plasma clearance for DPZ in human (10 L/h) [27].

Finally, the DPZ retained into nasal mucosa was investigated by extracting the drug from the tissue after the permeation studies. The tissues were washed with distilled water once removed from the diffusion cells at the end of the permeation experiments. The DPZ was recovered by ultrasound-assisted extraction using 1 mL of methanol for 20 min, and samples were analyzed by HPLC after filtration, leading to the drug retained in the tissue (Q_ret_) expressed as (µg DPZ/g tissue/cm^2^).

## Figures and Tables

**Table 1:** Composition formula of donepezil-loaded gels, expressed as percentage.

Ingredients (% *w*/*w*)	DPZ-CGel	DPZ-PGel_1_	DPZ-PGel_2_
DPZ	6.25 mg/mL	6.25 mg/mL	6.25 mg/mL
Chitosan	1	-	-
Pluronic F-127	-	18	18
Transcutol^®^ P	20	20	-
N-Methylpyrrolidone	-	-	5
Water	59	62	77
Acid acetic solution	20	-	-

**Table 2:** Viscosity, thixotropy, rheological behavior, and mathematical fitting of chitosan and Pluronic F-127 formulations.

Formulas	25 °C				35 °C			
	Viscosity (mPa·s) at 50 s^−1^	Apparent Thixotropy (Pa/s)	Rheological Behavior	Mathematical Model	Viscosity (mPa.s) at 50 s^−1^	Apparent Thixotropy (Pa/s)	Rheological Behavior	Mathematical Model
DPZ-CGEL	487 ± 1.5	72.7	Pseudoplastic	Cross (r = 1)	335 ± 0.9	43.3	Pseudoplastic	Cross (r = 1)
DPZ-PGel_1_	336 ± 3.3	39.5	Pseudoplastic	Cross (r = 1)	1345 ± 16.6	441.4	Pseudoplastic	Cross (r = 1)
DPZ-PGel_2_	232 ± 0.9	15.3	Pseudoplastic	Cross (r = 1)	1112 ± 21.5	568.7	Pseudoplastic	Cross (r = 1)

**Table 3:** Stability studies of chitosan and Pluronic F-127 formulations at 25 ± 2 °C/65 ± 5% RH.

Parameters	DPZ-CGel (Days)		DPZ-PGel_1_ (Days)		DPZ-PGel_2_ (Days)	
	1	30	1	30	1	30
Appearance	Homogeneous and slightly yellowish	Homogeneous and slightly yellowish	Homogeneous and translucent	Homogeneous and translucent	Homogeneous and translucent	Homogeneous and translucent
pH	6.30 ± 0.09	6.18 ± 0.05	6.30 ± 0.09	6.22 ± 0.03	6.30 ± 0.09	6.39 ± 0.05
Drug content (%)	99.70 ± 0.06	99.70 ± 0.09	99.60 ± 0.03	99.30 ± 0.08	101.30 ± 0.05	99.7 ± 0.10

**Table 4:** Stability studies of chitosan and Pluronic F-127 formulations at 40 ± 2 °C/75 ± 5% RH.

Parameters	DPZ-CGel (Days)		DPZ-PGel_1_ (Days)		DPZ-PGel_2_ (Days)	
	1	30	1	30	1	30
Appearance	Homogeneous and slightly yellowish	Homogeneous and slightly yellowish	Homogeneous and translucent	Homogeneous and translucent	Homogeneous and translucent	Homogeneous and translucent
pH	6.30 ± 0.09	6.33 ± 0.03	6.30 ± 0.09	6.26 ± 0.08	6.30 ± 0.09	6.39 ± 0.07
Drug content (%)	99.70 ± 0.06	98.67 ± 0.05	99.60 ± 0.03	99.50 ± 0.06	101.30 ± 0.05	100.50 ± 0.15

**Table 5:** Permeation parameters of donepezil (DPZ) formulations through nasal mucosa.

Permeation Parameters	DPZ-CGel	DPZ-PGel1	DPZ-PGel2
J_ss_ (µg/(h/cm^2^))	0.83 ± 0.07 ^b,c^	8.76 ± 0.92 ^a,c^	3.54 ± 0.29
Tl (h)	0.45 ± 0.05 ^b,c^	0.34 ± 0.02	0.31 ± 0.03
P_2_ (1/h)	0.37 ± 0.04 ^b,c^	0.48 ± 0.05	0.54 ± 0.05
P_1_ (cm) x 10^3^	0.36 ± 0.04 ^b,c^	2.90 ± 0.35 ^a,c^	1.05 ± 0.18
K_p_ (cm/h) x 10^3^	0.13 ± 0.01 ^b,c^	1.40 ± 0.20 ^a,c^	0.57 ± 0.07
Css (µg/mL)	0.01 ± 0.00 ^b,c^	0.13 ± 0.02 ^a,c^	0.05 ± 0.00
Q_ret_ (µg drug/g tissue/cm^2^)	546.96 ± 53.81 ^b,c^	620.23 ± 59.34	518.74 ± 57.21

Results are expressed by mean ± SD (n = 6). Statistical analysis ANOVA (followed by Tukey’s Multiple Comparison Tests): ^a^ statistical differences with DPZ-CGel; ^b^ statistical differences with DPZ-PGel1, and ^c^ statistical differences with DPZ-PGel2.

**Table 6:** Mathematical models for flow curve (shear stress (τ) versus shear rate ($\dot{\gamma }$)).

Model	Equation
Newton	$\tau =\eta \times \dot{\gamma }$
Bingham	$\tau =\tau_{0}+\left(\eta \times \dot{\gamma }\right)$
Ostwald-de-Waele	$\tau =K\times {\dot{\gamma }}^{n}$
Herschel-Bulkley	$\tau =\tau_{0}+K\times {\dot{\gamma }}^{n}$
Casson	$\tau =\sqrt[{n}]{\tau_{0}{}^{n}+{\left(\eta_{0}\times \dot{\gamma }\right)}^{n}}$
Cross	$\tau =\dot{\gamma }\times \left[\frac{\eta_{\infty }+\left(\eta_{0}-\eta_{\infty }\right)}{1+{\left(\frac{\dot{\gamma }}{{\dot{\gamma }}_{0}}\right)}^{n}}\right]$

where: *τ* is the shear stress (Pa); $\dot{\gamma }$ is the shear rate (1/s); *η* is the dynamic viscosity (mPa·s); *τ*_0_ is the yield shear stress (Pa); *η*_0_ is the zero-shear rate viscosity; *η*p is a constant plastic viscosity (mPa·s); *η*_∞_ is the infinity shear rate viscosity; *n* is the flow index and *K* is the consistency index.

**Table 7:** Kinetic model equations used for the Donepezil release profile.

Kinetic Model	Equation
Zero Order	$Q_{t}=K_{0}\cdot t+Q_{\infty }\;$
Higuchi	$Q_{t}=K_{H}\;t^{\frac{1}{2}}\;$
First Order	$Q_{t}=Q_{\infty }\left(1-e^{-K_{f}\cdot t}\right)$
Korsmeyer-Peppas	$Q_{t}=K_{k}\;t^{n}\;$

where: $Q_{t}$ is the amount of released drug at time t; $Q_{\infty }$ is the maximum amount of release drug; $K_{0},\;K_{f},K_{H},K_{k}$ are the constants of release rate; t is time in hours; n is the exponent of release (related to the drug release mechanism) n≤0.43 (Fickian diffusion), 0.43 < n < 0.85 (abnormal transport) and ≥0.85 (case II transport; zero-order release kinetic).

## Data Availability

Data is contained within the article.

## References

[1] Aderibigbe B.A. (2018). In Situ-Based Gels for Nose to Brain Delivery for the Treatment of Neurological Diseases. Pharmaceutics.

[2] Agrawal M., Saraf S., Saraf S., Antimisiaris S.G., Chougule M.B., Shoyele S.A., Alexander A. (2018). Nose-to-Brain Drug Delivery: An Update on Clinical Challenges and Progress towards Approval of Anti-Alzheimer Drugs. J. Control. Release.

[3] De La Torre C., Ceña V. (2018). The Delivery Challenge in Neurodegenerative Disorders: The Nanoparticles Role in Alzheimer’s Disease Therapeutics and Diagnostics. Pharmaceutics.

[4] Cavanaugh S.E., Pippin J.J., Barnard N.D. (2014). Animal Models of Alzheimer Disease: Historical Pitfalls and A Path Forward. Altex.

[5] Madav Y., Wairkar S., Prabhakar B. (2019). Recent Therapeutic Strategies Targeting Beta Amyloid and Tauopathies in Alzheimer’s Disease. Brain Res. Bull..

[6] Li Q., He S., Chen Y., Feng F., Qu W., Sun H. (2018). Donepezil-Based Multi-Functional Cholinesterase Inhibitors for Treatment of Alzheimer’s Disease. Eur. J. Med. Chem..

[7] Jacobson S.A., Sabbagh M.N. (2008). Donepezil: Potential Neuroprotective and Disease-Modifying Effects. Expert Opin. Drug Metab. Toxicol..

[8] Pires P.C., Santos A.O. (2018). Nanosystems In Nose-to-Brain Drug Delivery: A Review of Non-Clinical Brain Targeting Studies. J. Control. Release.

[9] Md S., Bhattmisra S.K., Zeeshan F., Shahzad N., Mujtaba M.A., Srikanth Meka V., Radhakrishnan A., Kesharwani P., Baboota S., Ali J. (2018). Nano-Carrier Enabled Drug Delivery Systems for Nose to Brain Targeting for the Treatment of Neurodegenerative Disorders. J. Drug Deliv. Sci. Technol..

[10] Silva-Abreu M., Espinoza L.C., Halbaut L., Espina M., García M.L., Calpena A.C. (2018). Comparative Study of Ex Vivo Transmucosal Permeation of Pioglitazone Nanoparticles for the Treatment of Alzheimer’s Disease. Polymers.

[11] Casettari L., Illum L. (2014). Chitosan in Nasal Delivery Systems for Therapeutic Drugs. J. Control. Release.

[12] Espinoza L.C., Vacacela M., Clares B., Garcia M.L., Fabrega M.J., Calpena A.C. (2018). Development of a Nasal Donepezil-Loaded Microemulsion for Treatment of Alzheimer’s Disease: In Vitro and Ex Vivo Characterization. Cns Neurol. Disord.-Drug Targets.

[13] Sullivan D.W., Gad S.C., Julien M. (2014). A Review of the Nonclinical Safety of Transcutol®, a Highly Purified Form of Diethylene Glycol Monoethyl Ether (Degee) Used as a Pharmaceutical Excipient. Food Chem. Toxicol..

[14] Roche-Molina M., Hardwick B., Sanchez-Ramos C., Sanz-Rosa D., Gewert D., Cruz F.M., Gonzalez-Guerra A., Andres V., Palma J.A., Ibanez B. (2020). The Pharmaceutical Solvent N-Methyl-2-Pyrollidone (Nmp) Attenuates Inflammation through Kruppel-Like Factor 2 Activation to Reduce Atherogenesis. Sci. Rep..

[15] Ciurlizza C., Fernandez F., Calpena A.C., Lazaro R., Parra A., Clares B. (2014). Semisolid Formulations Containing Cetirizine: Human Skin Permeation and Topical Antihistaminic Evaluation in a Rabbit Model. Arch. Dermatol. Res..

[16] Espinoza L.C., Vera-Garcia R., Silva-Abreu M., Domenech O., Badia J., Rodriguez-Lagunas M.J., Clares B., Calpena A.C. (2020). Topical Pioglitazone Nanoformulation for the Treatment of Atopic Dermatitis: Design, Characterization and Efficacy in Hairless Mouse Model. Pharmaceutics.

[17] Warnken Z.N., Smyth H.D.C., Watts A.B., Weitman S., Kuhn J.G., Williams R.O. (2016). Formulation and Device Design to Increase Nose to Brain Drug Delivery. J. Drug Deliv. Sci. Technol..

[18] Lenaerts V., Triqueneaux C., Quartern M., Rieg-Falson F., Couvreur P. (1987). Temperature-Dependent Rheological Behavior of Pluronic F-127 Aqueous Solutions. Int. J. Pharm..

[19] Berenguer D., Alcover M.M., Sessa M., Halbaut L., Guillen C., Boix-Montanes A., Fisa R., Calpena-Campmany A.C., Riera C., Sosa L. (2020). Topical Amphotericin B Semisolid Dosage Form for Cutaneous Leishmaniasis: Physicochemical Characterization, Ex Vivo Skin Permeation and Biological Activity. Pharmaceutics.

[20] Mallandrich M., Fernandez-Campos F., Clares B., Halbaut L., Alonso C., Coderch L., Garduno-Ramirez M.L., Andrade B., Del Pozo A., Lane M.E. (2017). Developing Transdermal Applications of Ketorolac Tromethamine Entrapped in Stimuli Sensitive Block Copolymer Hydrogels. Pharm. Res..

[21] Sosa L., Calpena A.C., Silva-Abreu M., Espinoza L.C., Rincon M., Bozal N., Domenech O., Rodriguez-Lagunas M.J., Clares B. (2019). Thermoreversible Gel-Loaded Amphotericin B for the Treatment of Dermal and Vaginal Candidiasis. Pharmaceutics.

[22] Udeni Gunathilake T.M.S., Ching Y.C., Chuah C.H. (2017). Enhancement of Curcumin Bioavailability Using Nanocellulose Reinforced Chitosan Hydrogel. Polymers.

[23] Vildanova R., Lobov A., Spirikhin L., Kolesov S. (2022). Hydrogels on the Base of Modified Chitosan and Hyaluronic Acid Mix As Polymer Matrices for Cytostatics Delivery. Gels.

[24] Ikeda T., Ikeda K., Yamamoto K., Ishizaki H., Yoshizawa Y., Yanagiguchi K., Yamada S., Hayashi Y. (2014). Fabrication and Characteristics of Chitosan Sponge as a Tissue Engineering Scaffold. BioMed Res. Int..

[25] Clementino A., Batger M., Garrastazu G., Pozzoli M., Del Favero E., Rondelli V., Gutfilen B., Barboza T., Sukkar M.B., Souza S.A.L. (2016). The Nasal Delivery of Nanoencapsulated Statins–an Approach for Brain Delivery. Int. J. Nanomed..

[26] Wengst A., Reichl S. (2010). Rpmi 2650 Epithelial Model and Three-Dimensional Reconstructed Human Nasal Mucosa as In Vitro Models for Nasal Permeation Studies. Eur. J. Pharm. Biopharm..

[27] Espinoza L.C., Silva-Abreu M., Clares B., Rodriguez-Lagunas M.J., Halbaut L., Canas M.A., Calpena A.C. (2019). Formulation Strategies to Improve Nose-to-Brain Delivery of Donepezil. Pharmaceutics.

[28] Rajput A., Butani S. (2022). Donepezil Hcl Liposomes: Development, Characterization, Cytotoxicity, and Pharmacokinetic Study. AAPS PharmSciTech.

[29] Gangopadhyay A., Dandagi P.M., Sutar K.P. (2022). Development and Evaluation of Thermoreversible Ethosomal Gel of Donepezil Hydrochloride for Intranasal Delivery. J. Pharm. Innov..

[30] Gangane P., Kawtikwar P. (2020). Development of Donepezil Hydrochloride Loaded Gellan Gum Based Nasal Mucoadhesive Microspheres by Spray Drying Method. Indian J. Pharm. Educ. Res..

[31] Gangane P.S., Ghormare N.V., Mahapatra D.K., Mahajan N.M. (2020). Gellan Gum Assisted Fabrication and Characterization of Donepezil Hydrochloride Mucoadhesive Intranasal Microspheres. Int. J. Curr. Res. Rev..

[32] Patil R., Pawara D., Gudewar C., Tekade A. (2019). Nanostructured Cubosomes in an In Situ Nasal Gel System: An Alternative Approach for the Controlled Delivery of Donepezil Hcl to Brain. J. Liposome Res..

